# Prevalence of foot problems in people with inflammatory arthritis in Singapore

**DOI:** 10.1186/s13047-016-0169-y

**Published:** 2016-09-02

**Authors:** K. Carter, M. Lahiri, P. P. Cheung, A. Santosa, K. Rome

**Affiliations:** 1 Podiatry Department, Rehabilitation Centre, National University Health System Singapore, Singapore, Singapore; 2 Yong Loo Lin School of Medicine, National University of Singapore, Singapore, Singapore; 3 Division of Rheumatology, Department of Medicine, National University Health System Singapore, Singapore, Singapore; 4 Health and Rehabilitation Research Institute, Faculty of Health and Environmental Sciences, AUT University, Auckland, New Zealand

**Keywords:** Inflammatory arthritis, Podiatry, Foot pain, Foot impairment

## Abstract

**Background:**

Foot problems are highly prevalent in people with inflammatory arthritis reported from studies in the UK, Europe and New Zealand, but there is limited evidence from Southeast Asia. The study aim was to evaluate the prevalence of foot problems in people with inflammatory arthritis in Singapore.

**Methods:**

People with inflammatory arthritis were recruited from the rheumatology outpatient clinic of a tertiary hospital in Singapore. Disease and clinical characteristics included age, sex, disease duration, current blood tests and medications. The Leeds Foot Impact Scale was used to evaluate foot impairment/disability and the Modified Health Assessment Questionnaire was used to assess global function.

**Results:**

We recruited 101 people with inflammatory arthritis, of which 50 % were female. The majority of participants were Chinese (70 %). The mean (SD) age was 52 (15) years, and the mean (SD) disease duration was 9.3 (0.3) years. The most commonly reported inflammatory arthritic conditions were rheumatoid arthritis (46), gout (31) and spondyloarthritis (15 %). The mean (SD) of the total Leeds Foot Impact Scale was 17 (13) indicating moderate to severe levels of foot impairment and activity limitation. Over 80 of participants reported foot pain during the course of their condition, and 48 % reported current foot pain. Despite the high prevalence of foot pain, only 21 participants (21 %) had been referred to a podiatrist.

**Conclusion:**

This is the first study to investigate the prevalence of foot problems in people with inflammatory arthritis from Singapore. The majority of the participants reported foot problems, but had not been referred to a podiatry service.

**Electronic supplementary material:**

The online version of this article (doi:10.1186/s13047-016-0169-y) contains supplementary material, which is available to authorized users.

## Background

Foot problems associated with inflammatory arthritis (IA) are common, particularly in Rheumatoid arthritis (RA) [1, 2]. Other IA conditions such as gout, spondyloarthritis (Spa) and psoriatic arthritis (PA) also affect the foot [3–5]. However, our knowledge of the epidemiology of foot involvement is based on studies among Caucasians derived primarily from Western countries [2, 6–8] with data currently dominated by studies focusing predominantly on people with RA [9]. While the magnitude of foot impairments and related disability in IA is comparable to that reported in RA [5, 10], relatively few studies to date have focused on IA foot and ankle characteristics from a Southeast Asian population.

RA affects approximately 0.4 % to 0.8 % of the adult population worldwide [11]. It is less prevalent in mainland China and in Hong Kong, with a reported prevalence of 0.37 and 0.35 %, respectively [12–14]. In Singapore, RA is the most common form of IA, affecting about 1 % of the population, equivalent to an estimated 45,000 people [15].

Previous studies of Chinese people have been largely population-based prevalence surveys [13, 16]. These studies suggest that RA in Asians behaves differently from that in Caucasians, such as a relatively greater involvement of the wrist joint and a milder disease course [13, 17, 18]. However, IA-related foot problems can be inadequately understood or overlooked during rheumatology consultations [19]. The aim of this study was to identify the prevalence of foot problems in people with IA attending a rheumatology outpatient clinic in Singapore.

## Method

### Participants

Participants were recruited from a rheumatology outpatient clinic in Singapore between January 2015 and November 2015. Each inflammatory arthritic condition was based upon medical records and referred by the rheumatologists to the podiatric service. Participants were eligible if they were over 21 years old, rheumatologist-diagnosed inflammatory joint disease and with or without current foot pain. Those with cognitive impairment precluding ability to answer health-related questions accurately were excluded. A convenience sample of 100 participants was predetermined based on a previous study [7]. Each participant attending the clinic was asked to consent to a foot health assessment and to complete a questionnaire. All assessments were conducted by an experienced podiatrist. Ethics approval was obtained from the National Healthcare Group Domain Specific Review Board Singapore. All participants provided written informed consent prior to data collection.

Podiatry services in Singapore are provided at the 6 public hospitals and by a small private sector. Singapore has approximately 80 podiatrists currently working, with most of them employed by the public hospitals. A team of 8 podiatrists works in the hospital in this study. Patients mainly access podiatry services by referral from a doctor. Most referrals come from within the hospital, with a small number coming from private family doctors and patient self-referral. A referral by internal doctor affords a government subsidy for the patient, which reduces their treatment charges by 50 %. Direct referral by a private family doctor and self-referral incurs full non-subsidized charges. At the rheumatology outpatient clinic where the study was conducted there are 13 rheumatologists and 25–30 clinics per week of mixed IA caseloads. During the period of the study there was no formally integrated multidisciplinary service operating; referral to podiatry was dependent on referrals from individual doctors, their decision to do so based on their knowledge of allied health care services or the willingness of the patient to be referred.

### Clinical characteristics

Clinical characteristics included the type of IA, disease duration, current medications, erythrocyte sedimentation rate (ESR) and C-reactive protein (CRP). For those participants with a previous foot x-ray the presence of radiographic foot erosions was recorded by reviewing the radiology reports for presence of erosions as documented within the clinical records. Global pain was measured using a 100 mm Visual analogue scale (VAS). The Disease Activity Score in 28-joints using the ESR (DAS28-ESR) was calculated for those people with RA and a same-day ESR result [20]. Responses to the Modified Health Assessment Questionnaire (MHAQ) - a physical function status questionnaire used in the evaluation of a variety of rheumatic diseases - were also recorded [21]. The MHAQ asks participants to answer 8 questions, 1 in each of the 8 functional areas [21]. The MHAQ assesses the degree of difficulty experienced with undertaking specific tasks over the preceding week. MHAQ scores are converted to a range between 0 and 3; with 0 to 1 indicating mild/moderate functional impairment, 1.1 to 2 is moderate/severe, and 2.1 to 3 indicating severe/very severe impairment [22].

### Foot and ankle characteristics

Foot and ankle characteristics included the Leeds Foot Impact Scale (LFIS) - a disease specific scale for measuring the impact of foot disease. The LFIS is a self-completed questionnaire comprising 51 items in total, divided into two subscales: impairments/shoes (LFIS_IF_) and activities/participation (LFIS_AP_) [23]. Turner and Woodburn [24] reported a LFIS_IF_ score of >7 and LFIS_AP_ score of >10 as representing a high to severe level of foot impairment and disability.

The Foot posture index (FPI) was used to assess foot type [25]. The Structural index (SI) evaluated forefoot and rearfoot deformities [26]. SI forefoot scores of ≥10 and rearfoot scores of ≥4 indicate the presence of severe foot deformity [24]. The Manchester scale evaluated the severity of hallux valgus [27]. Participants Previous and current foot pain was recorded Experience of current and previous foot pain and previously seen a podiatrist were recorded as dichotomous responses. Foot lesions that foot ulceration were noted. All demographic and disease activity data were presented as means and standard deviations (SD), and foot assessments as numbers and percentages.

## Results

The demographic and clinical characteristics of all participants are shown in Table 1. Additional file 1 demonstrates the demographic and clinical characteristics of each inflammatory arthritic condition that included RA (*n* = 46, 46), gout (*n* = 31, 31), Spa (*n* = 15, 15), PA (*n* = 4, 4) and undifferentiated IA (*n* = 5, 5 %). We recruited 101 participants, the majority of participants being Chinese women with a mean (SD) age of 52 (15) years. The majority of participants with RA were women (*n* = 37, 80) and men with gout (*n* =25, 81 %). The most commonly reported IA conditions were RA (*n* = 46, 46), gout (*n* = 31, 31) and spondyloarthritis (*n* = 15, 15 %). The mean (SD) disease duration across all IA conditions was 9.3 (0.3) years. The mean (SD) for BMI of 30.7 (5.0) Kg/m^2^ in gout was high compared to the other IA conditions. The MHAQ found mild overall functional impairment with a mean (SD) score of 0.25 (0.36). Blood markers (CRP and ESR) indicated high levels of inflammation in the 41 participants with RA.

**Table 1:** Demographic and clinical characteristics

Age, years	52.0 (14.5)
Women, n (%)	51 (50 %)
Ethnicity, n (%)	
Chinese	70 (69 %)
Malay	11 (11 %)
Indian	15 (15 %)
Caucasian	0 (0 %)
Other	5 (5 %)
Body Mass Index, Kg/m^2^	27.2 (5.4)
Smokers, n (%)	14 (14 %)
Disease duration, years	9.3 (0.3)
Disease type, n (%)	
• Rheumatoid arthritis	46 (46 %)
• Gout	31 (31 %)
• Spondyloarthritis	15 (15 %)
• Psoriatic arthritis	4 (4 %)
• Undifferentiated inflammatory arthritis	5 (5 %)
Medications, n (%)	
• Methotrexate	42 (60 %)
• Combination DMARD therapy (≥2 DMARDs)	52 (74 %)
• Biologics	4 (6 %)
• Prednisone	40 (40 %)
Other medications, n (%)	
NSAID, n (%)	49 (49 %)
Diabetes Mellitus, n (%)	12 (12 %)
Patient global VAS (VAS 0–100), mm	26 (26)
Tender (28) joint count	1.8 (2.8)
Swollen (28) joint count	1.3 (2.1)
DAS28-ESR score	3.54 (1.1)
ESR, mm/h	31.6 (21.2)
CRP, mg/L	27.4 (32.2)
mHAQ score	0.25 (0.36)

Data presented as mean (SD) unless specified

*DMARD* Disease modifying anti-rheumatic drug, *NSAID* Non steroidal anti-inflamamtory drugs, *VAS* Visual analogue scale, *ESR* Erythrocyte sedimentation rate, *CRP* C-reactive protein, *DAS-28* disease activity score in 28 joints

The foot and ankle characteristics are summarized in Table 2 with Additional file 1 demonstrating each specific IA condition. Over 80 % of participants (*n* = 81) reported having experienced foot pain during the course of their disease, with over 95 % of participants with gout reporting previous foot pain. Nearly 50 % of participants (*n* = 48) reported current foot pain, of which 45 (45 %) reported daily foot pain. Participants with RA reported current foot pain (*n* =28, 61 %), whereas only 7 (23 %) participants with gout reported current foot pain. Only 21 % participants had been referred to a podiatrist.

**Table 2:** Foot and ankle characteristics

Foot erosion on radiograph, n (%)	19 (40 %)^a^
Previous foot surgery, n (%)	9 (9 %)
Presence of current foot pain, n (%)	48 (48 %)
Previous foot pain, n (%)	81 (81 %)
Current foot ulceration, n (%)	1 (1 %)
Structural Index	
Forefoot score	4.9 (4.2)
Rearfoot score	3.5 (3.3)
Total Structural Index	8.4 (6.2)
Foot Posture Index foot-type	4 (6)
Callus Patterns:	
Forefoot, n (%)	12 (12 %)
Rearfoot, n (%)	0 (0 %)
Toes, n (%)	6 (6 %)
Severity of bunion, n (%)	
Stage 1	48 (48 %)
Stage 2	25 (25 %)
Stage 3	18 (18 %)
Stage 4	10 (10 %)
Has seen a podiatrist before, n (%)	21 (21 %)
FIS_TOTAL_ score	17 (13)
FIS_IF_ subscale score	7 (5)
FIS_AP_ subscale score	10 (9)

Data presented as mean (SD) unless specified

*FIS*_*TOTAL*_ Foot Impact Score total, *FIS*_*IF*_ Foot Impact Score foot impairment/footwear restriction, *FIS*_*AP*_ Foot Impact Score activity limitation/participation restriction

^a^48 participants underwent foot X-rays

The total mean (SD) of the LFIS was 17 (13). The mean (SD) for the LFIS_IF_ was 7 (7) and the LFIS_AP_ 10 (9) indicating moderate to severe levels of foot impairment and activity limitation. Participants with RA recorded higher levels of foot impairment and disability. The mean (SD) SI forefoot score of 4.9 (4.2) and the SI rearfoot score of 3.5 (3.3) demonstrated moderate levels of foot deformity. All participants with PA (*n* = 4, 100 %) were found to have high levels of rearfoot deformity. The FPI demonstrated a mean (SD) score of 4 (6).

## Discussion

The study demonstrates that foot problems are highly prevalent in people with IA attending the rheumatology outpatient clinic in Singapore. This study is the first to report prevalence of IA-related foot problems in a Southeast Asian population. Compared to the proportions of the main ethnic groups in Singapore this study demonstrates a representative sample [28]. The demographic and clinical characteristics of this IA cohort were consistent with other epidemiological studies from the UK and New Zealand [1, 3, 29–31]. In the current study 81 % of participants reported having had foot pain. Previous studies evaluating foot involvement in RA report involvement in 56–100 % of people [2, 6, 7, 32–34].

Current foot pain was reported by 48 % of patients with IA, noting a high prevalence in people with RA. Pain is the commonest problem facing patients with IA both generally and specifically related to their feet [31]. We found the data to be lower than has been previously reported in people with RA [6] and gout [35]. The differences may be due to previous studies being conducted in Western countries compared to Asian countries with regard to social, cultural and ethnic compositions [36]. However, there is limited information specifically to the foot in people with gout and RA in Asian populations.

We found the DAS28 demonstrated moderate levels of disease activity and the raised ESR and CRP suggests increased levels of inflammation. However, the DAS28 does not provide a measure of foot involvement, thus patients may be at risk of ongoing joint damage if treatment decisions are made solely on the basis of the DAS [37]. Given that patients can report severe symptoms in their feet, and these general health tools omit the feet, it is vital that foot health assessment tools are used in combination with the general disease assessment tools.

There is evidence that early intervention for existing or potential foot problems can improve long-term outcomes [38, 39]. Previous studies suggest that for people with IA, the involvement of the feet, even to a mild degree, is a significant marker for future impaired mobility, functional incapacity and negative psychosocial impact [7, 40]. Following diagnosis of IA a referral to a podiatrist for baseline assessment, tailored foot health education, self-care advice and necessary intervention, is recommended [40, 41]. However, with over one third of participants with moderate to severe foot impairment and moderate levels of foot-specific pain and deformity, very few patients had actually been referred for podiatry assessment. Greater emphasis on raising awareness of foot problems and podiatry care for people with IA is required in Singapore. Guidelines strongly advocate referral to a podiatrist for essential foot health management [40, 41]. Integration of podiatry services within the rheumatology multidisciplinary team (MDT) could resolve unmet need of people with current or potential IA-related foot problems. Emerging evidence suggests that tight pharmacological control, in conjunction with MDT care, including podiatry, can be effective in the management of people with IA [38, 42]. Further work is required to improve access to podiatry for people with IA in Singapore. Future developments should include the integration of specialist podiatrists into the MDT with emphasis on improving the quality and timeliness of patient care.

We found that only 21 % of participants had been referred to podiatry services. The strongest barriers preventing uptake of podiatry services appear to be: financial constraints (even with a subsidy out-of-pocket payment at the point of care can vary considerably for each service and for each patient, and therefore the cost to the patient plays a major role in healthcare decisions), lack of patient and /or doctor awareness and understanding of the role of podiatry, and low priority given to allied-health interventions by patients. Similar barriers have previously been reported in Australia [3, 43].

Limitations of this study may potentially be a lack of external validity as people were recruited from one tertiary hospital in Singapore and therefore, a true prevalence of foot problems is unknown. The sample of people attending the outpatient clinic may have resulted in selection bias. The study may also suffer from recall bias as some of the questions referred to events that occurred when the people were first diagnosed, past appointments and interventions and self-reporting of disease duration. We recruited from a mixed rheumatology caseload and grouped different IA conditions together for analysis, whereas previous studies have focused on a single condition such as RA [6, 7] or gout [35]. Although our findings are a true representation of the current service in Singapore, using a heterogeneous cohort potentially limits study comparison, analysis of specific differences and generalizability of findings.

The Leeds Foot Impact Scale was developed specifically to assess the rheumatoid foot, and has demonstrable measurement properties, such as reliability, construct validity, responsiveness, and wide applicability for the evaluation of the impact of disease on the feet [44]. However, anecdotal evidence from the current study identified that participants found it difficult to complete. Although there were no non-responses to the use of Leeds Foot Impact Scale, we observed that a minority of people were unable to comprehend the wording of the questions, especially if English was not their first language. Multiple generic and disease-specific foot scales are available in the English language [44]. Others such as the Foot Function Index, a generic foot scale, has undergone successful cross-cultural validation in several languages including Dutch, German and Taiwan Chinese [45–47]. The Leeds Foot Impact Scale is disease-specific, has been translated to Dutch, German and Hungarian [48]. Singapore’s majority population is Chinese and many older Singaporeans are not sufficiently proficient in the English language to enable questionnaire data to be collected without the aid of a translator. Additional work is required to validate a translated foot-specific patient reported outcome measure in simplified Chinese to facilitate further research into IA-related foot pain in Asian communities. Validated outcome tools that are cross-culturally invariant will also provide opportunity for wider international collaboration and comparison between populations.

## Conclusion

In conclusion, this is the first study to identify foot problems and uptake of podiatry services in people with IA in Singapore. The uptake of the podiatry service was poor and there is a need for foot-specific patient reported outcome measure in simplified Chinese to facilitate further research into IA-related foot pain, impairment and disability in Asian communities. The study highlights an unmet need for podiatry involvement and lack of compliance with international guidelines.

## Additional file

Additional file 1: Table S1. Demographic and clinical characteristics for each inflammatory condition. Data presented as mean (SD) unless specified. **Table S2.** Foot and ankle characteristics for each inflammatory condition. Data presented as mean (SD) unless specified. (DOCX 28 kb)

## Abbreviations

IA: Inflammatory arthritis
RA: Rheumatoid arthritis
Spa: Spondyloarthritis
PA: Psoriatic arthritis
Undifferentiated IA: Undifferentiated inflammatory arthritis
ESR: Erythrocyte sedimentation rate
CRP: C-reactive protein
DAS28-ESR: Disease activity Score in 28-joints using the ESR
MHAQ: Modified Health Assessment Questionnaire
LFIS: Leeds Foot Impact Scale
LFIS_TOTAL_: Leeds foot impact score total
LFIS_IF_: Leeds foot impact score foot impairment/footwear restriction
LFIS_AP_: sLeeds foot impact score activity limitation/participation restriction
FPI: Foot posture index
SI: Structural index
SD: Standard deviations
DMARD: Disease modifying anti-rheumatic drug
NSAID: Non-steroidal anti-inflammatory drugs
VAS: Visual analogue scale
MDT: Multidisciplinary team

## References

[1] Juarez M, Price E, Collins D, Williamson L (2010). Deficiencies in provision of integrated multidisciplinary podiatry care for patients with inflammatory arthritis: a UK district general hospital experience. Foot.

[2] Grondal L, Tengstrand B, Nordmark B, Wretenberg P, Stark A (2008). The foot: still the most important reason for walking incapacity in rheumatoid arthritis: distribution of symptomatic joints in 1,000 RA patients. Acta Orthop.

[3] Brenton-Rule A, Hendry G, Barr G, Rome K (2014). An evaluation of seasonal variations in footwear worn by adults with inflammatory arthritis: a cross sectional observational study using a web-based survey. J Foot Ankle Res.

[4] Roddy E, Muller S, Rome K, Chandratre P, Hider S, Richardson J (2015). Foot problems in people with gout in primary care: baseline findings from a prospective cohort study. J Foot Ankle Res.

[5] Hyslop E, McInnes IB, Woodburn J, Turner DE (2010). Foot problems in psoriatic arthritis: high burden and low care provision. Ann Rheum Dis.

[6] Otter S, Lucas K, Springett K, Moore A, Davies K, Cheek L (2010). Foot pain in rheumatoid arthritis prevalence, risk factors and management: an epidemiological study. Clin Rheumatol.

[7] Rome K, Gow PJ, Dalbeth N, Chapman JM (2009). Clinical audit of foot problems in patients with rheumatoid arthritis treated at Counties Manukau District Health Board, Auckland, New Zealand. J Foot Ankle Res.

[8] Koh ET, Tan JW, Thong BY, Teh CL, Lian TY, Law WG (2012). Major trends in the manifestations and treatment of rheumatoid arthritis in a multiethnic cohort in Singapore. Rheumatol Int.

[9] Lansdown N, Brenton-Rule A, Carroll M, Rome K (2015). Perceived barriers to the management of foot health in patients with rheumatic conditions. J Foot Ankle Res.

[10] Turner D, Hyslop E, Barn R, McInnes IB, Steultjens MPM, Woodburn J (2013). Metatarsophalangeal joint pain in psoriatic arthritis: a cross-sectional study. Rheumatology.

[11] Alamanos Y, Voulgari PV, Drosos AA (2006). Incidence and prevalence of rheumatoid arthritis, based on the 1987 American College of Rheumatology criteria: a systematic review. Semin Arthritis Rheum.

[12] Lau E, Symmons D, Bankhead C, MacGregor A, Donnan S, Silman A (1993). Low prevalence of rheumatoid arthritis in the urbanized Chinese of Hong Kong. J Rheumatol.

[13] Xiang YJ, Dai SM (2009). Prevalence of rheumatic diseases and disability in China. Rheumatol Int.

[14] Mok CC, Tam LS, Chan TH, Lee GKW, Li EKM, Hong Kong Society of Rheumatology (2011). Management of rheumatoid arthritis: consensus recommendations from the Hong Kong Society of Rheumatology. Clin Rheumatol.

[15] SingHealth. Rheumatoid arthritis. https://www.singhealth.com.sg/PatientCare/ConditionsAndTreatments/Pages/Rheumatoid-Arthritis.aspx. Accessed 1 Dec 2015.

[16] Zeng QY, Chen R, Darmawan J, Xiao ZY, Chen SB, Wigley R (2008). Rheumatic diseases in China. Arthritis Res Ther.

[17] Hoe J, Koh DR (1992). Rheumatoid arthritis in Singapore: carpal predominance and lower incidence of erosive changes. Ann Acad Med Singapore.

[18] Veerapen K, Mangat G, Watt I, Dieppe P (1993). The expression of rheumatoid arthritis in Malaysian and British patients: a comparative study. Br J Rheumatol.

[19] Rome K, Chapman J, Williams AE, Gow P, Dalbeth N (2010). Podiatry services for patients with arthritis: an unmet need. NZ Med J.

[20] Prevoo M, Van’t Hof MA, Kuper HH, Van Leeuwen MA, Van de Putte LBA, Van Riel P (1995). Modified disease activity scores that include twenty-eight-joint counts. Development and validation in a prospective longitudinal study of patients with rheumatoid arthritis. Arthritis Rheum.

[21] Pincus T, Summey JA, Soraci SA, Wallston KA, Hummon NP (1983). Assessment of patient satisfaction in activities of daily living using a modified Stanford health assessment questionnaire. Arthritis Rheumatol.

[22] Anderson J, Sayles H, Curtis J, Wolfe F, Michaud K (2010). Converting modified health assessment questionnaire (HAQ), multidimensional HAQ and HAQII scores into original HAQ scores using models developed with a large cohort of rheumatoid arthritis patients. Arthritis Care Res.

[23] Helliwell PS, Allen N, Gilworth G, Redmond A, Slade A, Tennant A, Woodburn J (2005). Development of a foot impact scale for rheumatoid arthritis. Arthritis Rheumatol.

[24] Turner D, Woodburn J (2008). Characterising the clinical and biomechanical features of severely deformed feet in rheumatoid arthritis. Gait Posture.

[25] Redmond AC, Crane YZ, Menz HB (2008). Normative values for the foot posture index. J Foot Ankle Res.

[26] Platto MJ, O’Connell PG, Hicks JE, Gerber LH (1991). The relationship of pain and deformity of the rheumatoid foot to gait and an index of functional ambulation. J Rheumatol.

[27] Garrow AP, Papageorgiou A, Silman AJ, Thomas E, Jayson MIV, Macfarlane GJ (2001). The grading of Hallux Valgus. J Am Podiatr Med Assoc.

[28] Singapore Department of Statistics. Census of population. 2015. http://www.singstat.gov.sg/docs/default-source/default-document-library/publications/publications_and_papers/reference/sif2016.pdf. Accessed 30 Aug 2016.

[29] Helliwell P (2003). Lessons to be learned: review of a multidisciplinary foot clinic in rheumatology. Rheumatology.

[30] Williams AE, Bowden AP (2004). Meeting the challenge for foot health in rheumatic diseases. Foot.

[31] Rome K, Erikson K, Ng A, Gow PJ, Sahid H, Williams AE (2013). A new podiatry service for patients with arthritis. NZ Med J.

[32] Matricali G, Bonen A, Verduyckt J, Taelman V, Verschueren P, Sileghem A (2006). The presence of forefoot problems and the role of surgery in patients with rheumatoid arthritis. Ann Rheum Dis.

[33] Shi K, Tomita T, Hayashida K, Owaki H, Ochi T (2000). Foot deformities in rheumatoid arthritis and relevance of disease severity. J Rheumatol.

[34] Borman P, Ayhan F, Tuncay F, Sahin M (2012). Foot problems in a group of patients with rheumatoid arthritis: an unmet need for foot care. Open Rheumatol J.

[35] Rome K, Frecklington M, McNair P, Gow P, Dalbeth N (2012). Foot pain, impairment, and disability in patients with acute gout flares: a prospective observational study. Arthritis Care Res.

[36] Kelli A (2010). Racial and ethnic disparities in osteoarthritis phenotypes. Curr Opin Rheumatol.

[37] Bakker MF, Jacobs JW, Kruize AA, van der Veen MJ, van Booma-Frankfort C, Vreugdenhil SA (2012). Misclassification of disease activity when assessing individual patients with early rheumatoid arthritis using disease activity indices that do not include joints of feet. Ann Rheum Dis.

[38] Woodburn J, Hennessy K, Steultjens M, McInnes, Turner D (2010). Looking through the ‘window of opportunity’: is there a new paradigm of podiatry care on the horizon in early rheumatoid arthritis?. J Foot Ankle Res.

[39] Gossec L, Pavy S, Pham T, Constantin A, Poiraudeau S, Combe B (2006). Non-pharmacological treatments in early rheumatoid arthritis: clinical practice guidelines based on published evidence and expert opinion. Joint Bone Spine.

[40] Arthritis and Musculoskeletal Alliance. Standards of care for people with foot health problems and inflammatory arthritis. 2004. http://www.arma.uk.net/resources/standards-of-care. Accessed 12 Dec 2015.

[41] Scottish Intercollegiate Guideline Network (2000). Management of early rheumatoid arthritis. A National Clinical Guideline.

[42] Vliet Vlieland TP, Pattison D (2009). Non-drug therapies in early rheumatoid arthritis. Best Pract Res Clin Rheumatol.

[43] Hendry G, Gibson K, Pile K, Taylor L, Du Toit V, Burns J, Rome K (2013). “They just scraped off the calluses”: a mixed methods exploration of foot care access and provision for people with rheumatoid arthritis in south-western Sydney, Australia. J Foot Ankle Res.

[44] van der Leeden M, Steultjens MP, Terwee CB, Rosenbaum D, Turner D, Woodburn J, Dekker J (2008). A systematic review of instruments measuring foot function, foot pain, and foot-related disability in patients with rheumatoid arthritis. Arthritis Care Res.

[45] Naal FD, Impellizzeri FM, Huber M, Rippstein PF (2008). Cross-cultural adaptation and validation of the foot function index for use in German-speaking patients with foot complaints. Foot Ankle Int.

[46] Kuyvenhoven MM, Gorter KJ, Zuithoff P, Budiman-Mak E, Conrad KJ, Post MW (2002). The foot function index with verbal rating scales (FFI-5 pt): a clinimetric evaluation and comparison with the original FFI. J Rheumatol.

[47] Wu SH, Liang HW, Hou WH (2008). Reliability and validity of the Taiwan Chinese version of the foot function index. J Formos Med Assoc.

[48] Woodburn J, Turner DE, Rosenbaum D, Balint G, Korda J, Ormos G (2012). Adaptation and cross cultural validation of the foot impact scale for rheumatoid arthritis using Rasch analysis. Arthritis Care Res.

