# Supplementary material for: Prevalence of foot problems in people with inflammatory arthritis in Singapore
Source: J Foot Ankle Res. 2016 Sep 2;9(1):37. doi: 10.1186/s13047-016-0169-y (PMC5010761; doi:10.1186/s13047-016-0169-y)
Supplement: Additional file 1: Table S1. — Demographic and clinical characteristics for each inflammatory condition. Data presented as mean (SD) unless specified. Table S2. Foot and ankle characteristics for each inflammatory condition. Data presented as mean (SD) unless specified. (DOCX 28 kb) [file 13047_2016_169_MOESM1_ESM.docx]

**Supplementary File**

Table 1: Demographic and clinical characteristics for each inflammatory condition. Data presented as mean (SD) unless specified.

| **Variable** | **RA (n=46)** | **Gout (n=31)** | **Spa (n=15)** | **PA (n=4)** | **UIA (n=5)** |
| --- | --- | --- | --- | --- | --- |
| Age, years | 56.1 (11.9) | 50.0 (16.2) | 43.2 (15.7) | 54.0 (13.7) | 51.2 (11.2) |
| Women, n (%) | 37 (80%) | 6 (19%) | 5 (33%) | 1 (25%) | 3 (60%) |
| Ethnicity, n (%)  Chinese  Malay  Indian  Caucasian  Other | 29 (63%)  5 (11%)  9 (20%)  0 (0%)  3 (7%) | 24 (77%)  3 (10%)  3 (10%)  0 (0%)  0 (0%) | 10 (67%)  3 (10%)  1 (3%)  0 (0%)  1 (3%) | 3 (75%)  0 (0%)  1 (25%)  0 (0%)  0 (0%) | 4 (80%)  0 (0%)  1 (20%)  0 (0%)  0 (0%) |
| Body Mass Index, Kg/m^2^ | 25.8 (4.8) | 30.7 (5.0) | 25.1 (3.7) | 27.2 (6.7) | 23.6 (4.2) |
| Smokers, n (%) | 4(4%) | 10 (29%) | 0 (0%) | 0 (0%) | 0 (0%) |
| Disease duration, years | 7.6 (0.3) | 11.8 (10.5) | 12.9 (12.3) | 11.5 (0.5) | 2.6 (2.2) |
| Diabetes Mellitus, n (%) | 4 (4%) | 7 (23%) | 0 (0%) | 1 (25%) | 0 (0%) |
| Patient global VAS, mm | 35.1 (26.0) | 13.1 (27.1) | 28.0 (28.9) | 31.3 (16.0) | 26.0 (19.5) |
| Tender (28) joint count | 2.3 (2.9) | 0.9 (1.9) | 2.0 (3.8) | 2.5 (2.4) | 2.4 (2.3) |
| Swollen (28) joint count | 1.7 (2.3) | 0.7 (1.4) | 0.6 (1.4) | 4.0 (3.3) | 1.4 (2.1) |
| DAS28-ESR score | 3.6 (0.7) | NA | 3.5 (1.4) | NA | NA |
| ESR, mm/hr | 32.7 (21.3) | NA | 2.4 (1.1) | NA | 15.3 (5.2) |
| CRP, mg/L | 37.9 (36.9) | NA | 7.3 (2.5) | NA | NA |
| mHAQ score | 0.26 (0.37) | 0.23 (0.34) | 0.37 (0.40) | 0.19 (0.21) | 0.02 (0.01) |

Table 2: Foot and ankle characteristics for each inflammatory condition. Data presented as mean (SD) unless specified.

| **Variable** | **RA (n=46)** | **Gout (n=31)** | **Spa (n=15)** | **PA (n=4)** | **UIA (n=5)** |
| --- | --- | --- | --- | --- | --- |
| Foot erosion on radiograph, n (%) | 18 (45%) | 9 (29%) | 0 (0%) | 2 (50%) | 0 (0%) |
| Presence of current foot pain, n (%) | 28 (61%) | 7 (23%) | 8 (53%) | 2 (50%) | 3 (80%) |
| Previous foot pain, n (%) | 32 (70%) | 30 (97%) | 12 (80%) | 4 (100%) | 2 (40%) |
| Current foot ulceration, n (%) | 1 (2%) | 1 (3%) | 0 (0%) | 0 (0%) | 0 (0%) |
| Structural Index  Forefoot score  Rearfoot score  Total Structural Index | 6 (4)  4 (3)  10 (6) | 4 (3)  4 (3)  7 (6) | 4 (4)  2 (5)  5 (6) | 4 (1)  9 (4)  13 (5) | 1 (1)  2 (3)  3 (3) |
| Foot Posture Index foot-type | 6 (5) | 2 (7) | 3 (5) | 8 (3) | 2 (5) |
| Severity of bunion, n (%)  Stage 1  Stage 2  Stage 3  Stage 4 | 18 (39%)  12 (26%)  9 (20%)  7 (15%) | 18 (58%)  7 (23%)  2 (6%)  4 (13%) | 9 (60%)  5 (33%)  1 (7%)  0 (0%) | 3 (75%)  1 (25%)  0 (0%)  0 (0%) | 2 (40%)  1 (20%)  2 (40%)  0 (0%) |
| FIS_TOTAL_ score | 19 (13) | 16 (12) | 15 (13) | 13 (20) | 12 (8) |
| FIS_IF_ subscale score | 8 (5) | 7 (5) | 7 (5) | 6 (8) | 6 (4) |
| FIS_AP_ subscale score | 11 (9) | 9 (9) | 9 (9) | 7 (13) | 6 (6) |
